# Relationships between followers’ behaviors and job satisfaction in a sample of nurses

**DOI:** 10.1371/journal.pone.0185905

**Published:** 2017-10-05

**Authors:** Paola Gatti, Chiara Ghislieri, Claudio G. Cortese

**Affiliations:** Department of Psychology, Università degli Studi di Torino, Turin, Italy; Kyoto University, JAPAN

## Abstract

The study investigated two followership behaviors, followers’ active engagement and followers’ independent critical thinking, and their relationship with job satisfaction in a sample of nurses. In addition, the study also considered a number of control variables and classical job demands and job resources—workload and emotional dissonance for job demands, and meaningful work for job resources—which have an impact on well-being at work. A paper-and-pencil questionnaire was administered to 425 nurses in an Italian hospital, and a hierarchical multiple regression was used to test the hypotheses. In addition to the job demands and job resources considered, followers’ active engagement had a significant impact on job satisfaction. Moreover, it showed a significant linear and curvilinear relationship with the outcome variable. Followers’ independent critical thinking has a non significant relationship with job satisfaction, confirming the mixed results obtained in the past for this dimension. These findings bore out the importance of analyzing followers’ behaviors as potential resources that people can use on the job to increase their own well-being. Looking at followers not just as passive recipients but as active and proactive employees can also benefit the organization.

## Introduction

The importance of followership, and its relevance for the organization, are clear to many authors who describe followership as a precondition for organizations’ success [1–4]. Notwithstanding this, followership behaviors—that is, “behaviors of individuals acting in relation to a leader(s)” ([5], p. 545)–have received scant attention both in the literature, and in empirical studies. By contrast the relationship with the leader to which followership behaviors are clearly linked, and especially the construct of leader-member exchange, has been extensively investigated, with thousands of publications.

The main purpose of this study, which was carried out with a sample of nurses, is thus to investigate followership behaviors as resources that can increase job satisfaction—one of the classical outcomes for leader-member exchange (LMX) [6]–in a linear as well as in a curvilinear way.

In so doing, we draw inspiration from the work of Kelley [1], and thus distinguished between followers’ active engagement (F.AE), that is the tendency to participate actively and take initiative especially in the relationship with the leader, and followers’ independent critical thinking (F.ICT), that is the behavior of offering constructive criticism and thinking in an innovative way. We investigated these behaviors in the light of the job demands-resources model (JD-R model) [7], a framework which has already been applied to study the nursing profession (e.g., [8]) and which is widely used in investigations of well-being at work where it distinguishes the impacts of two different sources of well-being and motivation, resources and demands respectively [7].

Job satisfaction (JS) is a variable which can be analyzed in this framework, and has been described in a recent paper by Bakker as one of the four central dimensions linked to well-being at work [9]. Several resources and demands were shown to have an influence on this outcome. In this study, we investigated the impact that a resource such as meaningful work (MW) and demands such as workload (WL) and emotional dissonance (ED) can have on JS. These variables were chosen thinking of the characteristics of our sample of nurses. The nursing profession involves work that can be considered a mission achieved by providing patient-oriented care [10] and that, at the same time, can put the worker in emotionally complicated situations. It has thus been recognized as a “demanding profession” [11].

Among resources, we also studied positive behaviors in the follower role, dimensions which have not yet been considered in the major studies on the JD-R model [7, 12–14]. As these behaviors are closely linked with the leader-follower relationship, and LMX has been shown to have curvilinear relationships with important outcome variables, especially those linked to stress [15], the aims of this paper included investigating the curvilinear relationship that F.AE and F.ICT could have with JS, in addition to the linear relationships between them. We thus hypothesized that for high F.AE values, the perception of JS increases curvilinearly and that for high F.ICT the outcome variable diminishes in the same way, even after controlling for all the other variables in our study. Summarizing, this work bridges a gap in the literature by investigating followership behaviors as resources that can increase JS, in a linear as well as in a curvilinear way.

## Followers’ behaviors as job resources

Leadership styles and the quality of the relationship with one’s own leader have a crucial role in the nursing profession, as a recent special issue of the *Journal of Nursing Management* [16] proved. Notwithstanding this, positive followership behaviors, linked to the relationship between leader and follower and to how the follower role is construed, have not yet been included in lists of resources having an influence on well-being and motivation. Since job resources were defined as “those physical, social, or organizational aspects of the job” ([7], p. 501) that can be instrumental in achieving work goals, followers’ behaviors could satisfy this description.

Recently, Kelley has been credited as the author who initiated the role-based approach to followership in the review on the topic by Uhl-Bien and colleagues [17]. Kelley described the ideal follower as someone who takes part in a joint process of achieving a common purpose [1, 4, 18]. His typology uses two quadrants: passive–active and dependent–independent, and describes “exemplary followers” as actively engaged and capable of exhibiting courageous conscience [1]. In Kelley’s work, followership is operationalized along two main dimensions: a) Active Engagement (F.AE), or the propensity to take initiative, participate actively and be self-starters, especially in the relationship with the leader; b) Independent Critical Thinking (F.ICT), which consists in offering constructive criticism and showing the ability to think for oneself, with creativity and innovation, including toward one’s own leader. The topic of proactivity seems to clearly summarize the two behaviors described by Kelley, and the positive side of his two quadrants (independent and active). For instance, among proactive behaviors, we can state that voice behaviors—the “discretionary communication of ideas, suggestions, concerns, or opinions about work-related issues with the intent to improve organizational or unit functioning” ([19], p. 375)–could recall F.ICT even if the latter behavior is directed toward the leader.

Few studies have investigated the relationship between these two followership behaviors and the variables in their nomological network. In addition to the studies which analyzed F.AE and F.ICT in relation to their antecedents (e.g., [20, 21]), or the former behavior and its antecedents [22], Blanchard and colleagues [23], in their effort to validate Kelley’s scale [1], also investigated the relationships between the two dimensions and some potential outcomes, specifically well-being and motivational indicators at work. They found that F.AE was positively related to JS (intrinsic and extrinsic) and organizational commitment (affective and normative), whereas F.ICT was negatively related to normative organizational commitment and extrinsic job satisfaction. In the Italian validation of Kelley’s scale [24], a significant positive correlation was found between F.AE and JS, and a non-significant relationship between F.ICT and JS. Considering the definitions of the two behaviors, and their operationalization, which emphasize the positive side of F.AE and F.ICT, as well as the mixed results mentioned above, we hypothesize that:

*Hp*. *1*—F.AE and F.ICT have a positive relationship with JS. This relationship is stronger between F.AE and JS than between F.ICT and the outcome variable.

Moreover, in addition to linear relationships, several theories and models linked to well-being hypothesize curvilinear relationships of antecedents on their outcomes or, more generally, suggest that it is necessary to distinguish different ways of functioning for the antecedents (see for instance activation theory [25] and the vitamin model [26]).

Interest in investigating the potential curvilinear relationship between followers’ behaviors and JS has been sparked by several papers which showed that there is a curvilinear relationship between LMX and variables linked to work stress [15, 27, 28]. Stress outcomes are closely linked to JS, while followers’ behaviors are part (or are an aspect) of that exchange relationship that could show curvilinear effects if what is received by the follower seems excessive and hence very difficult to reciprocate [15].

For this reason as well, we hypothesize that F.AE—as a measure of the behavior of followers who describe themselves as highly involved in the job, proactive, self-confident and autonomous—could have a curvilinear relationship with JS. This curvilinear relationship would work as follows: for high levels of F.AE, JS will increase more than it would if the relationship were linear. Followers with high F.AE will perceive that they reciprocate what was received in the exchange with the leader more than adequately. In addition, proactivity and the belief in the legitimacy of high levels of personal initiative are characteristics which may have a close and curvilinear link with job satisfaction [29].

As for F.ICT, the curvilinear relationship is expected to have an inverted U-shape. Proactive behaviors can usually be a source of JS [30], since they can lead to success and high performance, but when they are closely linked to challenging non-required behaviors such as voice [31], they can have a more complicated relationship with satisfaction. Engaging in voice behaviors may involve an element of social risk [32] and so, for instance, the decision to adopt or avoid such behaviors hinges to a large extent on the relationship that followers have with their leader [33]. A similar mechanism could work for F.ICT, which has some similarity with voice behaviors, even if it is more directly oriented towards the leader. F.ICT could thus increase JS up to a certain level and, above this critical point, could even be harmful. Considering voice behaviors, curvilinear relationships have been effectively measured [34, 35]. Summarizing, we hypothesize that:

*Hp*. *2*—F.AE has a U-shaped curvilinear relationship with JS. Specifically, high levels of F.AE correspond to higher levels of JS than in a linear relationship.

*Hp*. *3*—F.ICT has an inverted-U relationship with JS. Specifically, high levels of F.ICT correspond to lower levels of JS than in a linear relationship.

## Job satisfaction in the framework of the JD-R model

Locke ([36], p. 1300) defined JS as “a pleasurable or positive emotional state resulting from the appraisal of one’s job or job experiences”. Though this construct is not traditionally a core concept for the JD-R model [7], a recent paper by Bakker and Oerlemans [9] considered JS as one of the four main constructs in this area (together with engagement, burnout and workaholism). The literature on JS and its determinants is very vast—with several meta-analytic studies, some of which deal specifically with nurses (e.g., [37, 38]) and with studies on nurses’ JS in Italy [39–41]–so that an exhaustive review would be practically impossible. We will thus concentrate here on the papers that analyze JS in the framework of the JD-R model, considering both resources and demands, with a focus on those relevant to the nursing profession.

As regards resources, using the framework of the JD-R model, Mauno, Kinnunen and Ruokolainen [42] showed that job control, family supportive climate and organization-based self-esteem increased JS; Nielsen, Mearns, Matthiesen and Eid [43] showed that psychosocial safety climate buffers the effect of risk perception on JS, while Hall, Dollard, Winefield, Dormann and Bakker [44] showed that this resource buffers the effect of depression on JS and engagement considered together.

In studies on nurses, the following resources were mentioned, for instance, as JS antecedents: opportunities for developing one’s own competence (opportunities for professional development, in Schaufeli and Taris [14]), social support [39, 45], and autonomy [46], which is confirmed as a significant predictor in a meta-analysis for the nursing profession [38].

This paper considers the job resource of meaningful work (MW), which can be defined as the perception of doing a meaningful job that enables people to express their potential and to achieve their purpose. It is described as “the point at which a person’s passions, strengths, and core values interact synergistically in his or her work” ([47], p. 1384). Work is thus meaningful when it has a purpose that is greater than the extrinsic outcomes that a person can obtain through it [48]. This construct can be considered as a component of a view of work that entails being able to reach a person’s transcendent dimension through it, an aspect which is particularly relevant in the healthcare sector [49, 50]. Also the classical Job Characteristics Model [51] emphasizes the link between the experienced meaningfulness of the work, as a psychological state influenced by the core job characteristics, and JS [52]. Considering the definition of MW and previous results, we thus hypothesize:

*Hp*. *4*—MW has a significant positive relationship with JS.

As regards demands, again using the JD-R model as a framework for the investigation, Nielsen and colleagues [43] showed that risk perception decreases JS.

Studies on nurses refer, for instance, to the following demands as JS antecedents: psychological demands and unfavorable work schedule [46], role conflict and qualitative demands [53], while unclear nurses’ responsibilities, poor leadership skills and discrimination at work, are among the antecedents mentioned in a qualitative study [54].

This study considered two job demands: workload (WL) and emotional dissonance (ED), both mentioned in descriptions of the JD-R model. WL was always present in the initial papers on the model written by its designers [12, 13], and is described as a general type of demand consisting of having too much work to do and not enough time to do it [55]. Even though it is a general job demand, a number of studies considering this variable for nurses found a negative relationship between WL and JS [56]. Qualitative studies on nurses also describe JS and WL as major concerns in this profession [57].

The second job demand investigated here is ED, a type of demand specific to people-oriented professions which consists of a discrepancy between felt and displayed emotions [58]. As Zapf and colleagues describe [59]: “Emotional dissonance occurs when an employee is required to express emotions that are not genuinely felt in the particular situation” (p. 375). By definition, this demand can be particularly relevant for human service professions [58]. For instance, Bakker and Heuven [58] showed that ED is linked to burnout both in a sample of nurses and in a sample of police officers. Considering the link between this demand and JS, Lewig and Dollard [60] found that ED has more power than other emotional demands to account for variance in emotional exhaustion and JS. We thus hypothesize that:

*Hp*. *5*—WL and ED have a significant negative relationship with JS.

**Fig 1:**
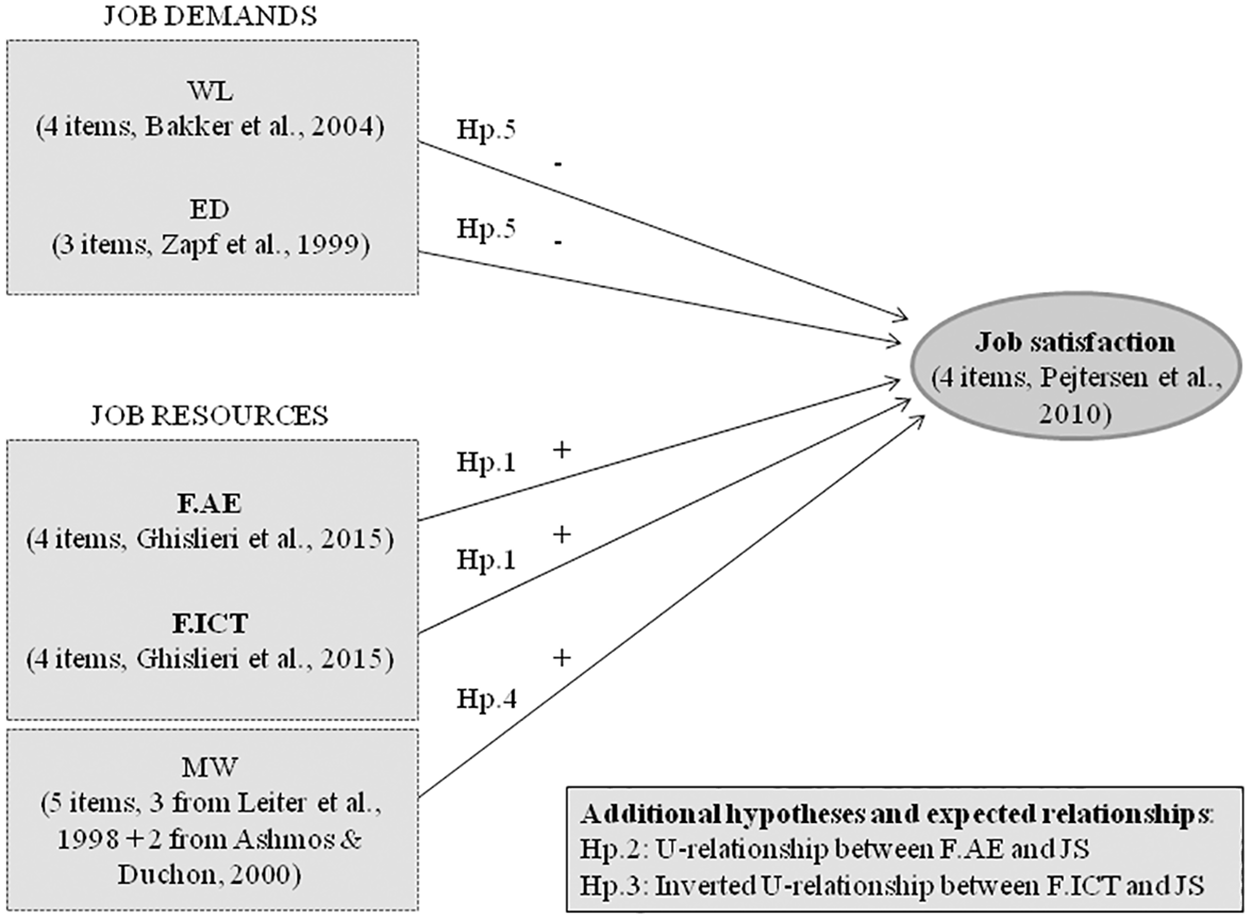
The study model. *Note*. The figure shows the model tested in the study with all the hypotheses and expected relationships between each demand/resource and the outcome of JS. WL = workload; ED = emotional dissonance; F.AE = follower’s active engagement; F.ICT = follower’s independent critical thinking; MW = meaningful work; JS = job satisfaction. The variable short name is followed by a description of how it was measured. Further details are given in the Measures section.

Fig 1 below summarizes the variables in our studies and their hypothesized relationships with JS, also listing the five hypotheses that the study tests. The two hypotheses which regard curvilinear relationships, and thus differ from the classical hypotheses in the JD-R model, are presented in a separate box.

## Procedure and participants

Participants consisted of 425 nurses (who were not head nurses or nurse managers, i.e., who were not in charge of groups) working in the same large hospital in a town in the North-West of Italy.

Paper and pencil questionnaires were distributed to all 559 nurses working in the hospital. As 442 questionnaires were collected by the researchers, the overall response rate was 79.1%. This very high percentage of responses was partly due to the detailed communication that potential participants received concerning the project. This sample was cleaned by deleting 17 questionnaires because of poor responses.

The study was carried out in line with the Helsinki Declaration [61], as well as Italian data protection legislation. It was authorized by the hospital nursing director. Since there was no medical treatment or other procedures that could cause psychological or social discomfort to participants, additional ethical approval was not required. To guarantee anonymity, respondents returned questionnaires in drop boxes, after sealing each of them in an envelope provided by researchers. A cover letter attached to the questionnaire described the measures taken to guarantee anonymity, the voluntary nature of participation for which no recompense was envisaged, and the guidelines for filling out the questionnaire. A statement at the end of the cover letter/information sheet clarified that respondents who filled in the questionnaire were thereby consenting to participate in the research project.

The socio-demographic characteristics of the 425-respondent sample are summarized in Table 1.

**Table 1:** Socio-demographic characteristics of the sample.

Dimension	Sample mean/percentage
**Gender**	86.6% women 13.4% men
**Age**	39.6 years (*SD* 8.18, from min = 23 to max = 60)
**Education level**	44.7% with a high school diploma or less 39.6% with a bachelor’s degree 15.7% with a master’s degree or other post-graduate qualification
**Type of contract (length)**	96.5% with permanent contract 3.5% with temporary contract
**Type of contract (hours worked)**	82.9% full-time contract 17.1% part-time contract
**Hours worked per week**	34.9 (*SD* 5.69, from min = 18 to max = 50)
**Tenure in organization**	15.6 years (*SD* 9.36, from min = 1 to max = 40)
**Length of employment**	17.8 years (*SD* 9.42, from min = 1 to max = 41)
**Working areas**	35.0% of respondents worked in medicine 23.8% in surgery 23.3% in intensive care 18.0% in the service area

Note. The service area, which is a standard part of Italian hospital organizations and deals with both inpatients and outpatients, comprises all healthcare services that deal with diagnosis, e.g., the radiology and blood testing services, and all the outpatient services for specific wards, such as the pain therapy outpatient program.

## Measures

In addition to a socio-demographic section, which also included questions about the control variables used in this investigation (gender, tenure in organization, hours worked per week), the questionnaire consisted of the following scales.

*Job satisfaction* (JS) was measured with the 4 items developed by Pejtersen, Kristensen, Borg and Bjorner [62], which were on a 5-point Likert scale (1 = very dissatisfied, 5 = very satisfied). An example item is: “How pleased are you with… the physical working conditions?”. Variance explained by the EFA solution (ML) was 51.4%, while Cronbach’s alpha was .80.

*Follower’s active engagement* (F.AE) was assessed using the short Italian version of The Power of Followership Scale by Kelley [1], developed and validated by Ghislieri, Gatti and Cortese [63]. F.AE was measured by 4 items on a 7-point Likert scale (0 = never, 6 = always). An example item is: “Do you take the initiative to seek out and successfully complete assignments that go above and beyond your job?”. Explained variance (ML, Oblimin) was 46.4%, while Cronbach’s alpha was .80.

*Follower’s independent critical thinking* (F.ICT) was assessed using the 4 items (0 = never, 6 = always) in the short followership scale validated by Ghislieri, Gatti and Cortese [63]. An example item is: “When your departmental chairperson asks you to do something that runs contrary to your professional or personal preferences, do you say ‘no’ rather than ‘yes’?”. Explained variance (ML) was 46.4%, while Cronbach’s alpha was .73.

*Meaningful work* (MW) was assessed using 5 items, and specifically 3 taken from the scale by Leiter, Harvie and Frizzell [64], which was developed for nurses, and 2 from the scale by Ashmos and Duchon [65]. The items were on a 7-point Likert response scale (1 = strongly disagree, 7 = strongly agree). An example item is: “The work I do is connected to what I think is important in life”. Explained variance (ML) was 56.9%, while Cronbach’s alpha was .87.

*Workload* (WL) was assessed with 4 items by Bakker, Demerouti and Verbeke [66], which were on a 5-point Likert scale (1 = never, 5 = always). An example item is: “Do you work under time pressure?”. Explained variance (ML) was 49.3%, while Cronbach’s alpha was .77.

*Emotional dissonance* (ED) was measured using 3 items by Zapf, Vogt, Seifert, Mertini and Isic [59], which were on a 6-point Likert scale (1 = never, 6 = always). An example item is: “How often does it occur in your job that you have to… Display emotions which do not correspond to inner feelings?”. Explained variance (ML) was 70.2%, while Cronbach’s alpha was .87.

## Analyses

In addition to the measure of Cronbach’s alpha, used to test for homogeneity and internal consistency [67], exploratory factor analysis (EFA, ML method of extraction) was performed on each scale and the factor scores were saved and used for the correlations and the hierarchical multiple regression. To verify that the items of the scales did not load on a scale other than the expected one, we also performed an EFA with all the items of interest, checking for eigenvalues greater than 1. Lastly, a hierarchical multiple regression was carried out to test the study hypotheses. In the last step of the multiple regression, we added the squared component of our two followership scales to test for curvilinear relationships. We used SPSS Statistics 22 for all these analyses.

## Results

The EFA (ML, Promax rotation) on the 24 items used to measure the investigated variables showed good results. As expected, six eigenvalues were greater than 1 and the average variance extracted was 54.6%. None of the items showed high loadings (greater than or equal to .3) on a different factor from the one it was developed to measure. Results of this preliminary check were thus satisfactory.

Table 2 shows means, standard deviations and intercorrelations of all variables. JS shows positive correlations with MW (r .41, *p* < .001), F.AE (r .21, *p* < .001) and F.ICT (r .10, *p* < .05), while negative correlations with ED (r -.29, *p* < .001) and WL (r -.14, *p* < .001). It has no significant correlations with the control variables.

**Table 2:** Means, standard deviations and intercorrelations of all variables.

	1.	2.	3.	4.	5.	6.	7.	8.	9.
1. JS	-								
2. Gender (1 = male)	.08	-							
3. Tenure in organiz.	.03	-.24**	-						
4. Hours worked	.01	.02	-.42**	-					
5. F.AE	.21**	.15**	-.10	.09	-				
6. F.ICT	.10*	-.01	-.01	.16**	.46**	-			
7. MW	.41**	.03	-.16**	.04	.29**	.19**	-		
8.WL	-.14**	.04	-.13**	.16**	.06	.05	-.01	-	
9. ED	-.29**	.05	.03	.06	-.06	-.11*	-.21**	.17**	-
*M*	3.33	-	15.6	34.9	3.36	3.66	5.18	3.32	3.05
*SD*	0.67	-	9.36	5.69	1.01	1.02	1.10	0.65	1.28

Note.

* *p* < .05;

** *p* < .01.

JS = job satisfaction; F.AE = follower’s active engagement; F.ICT = follower’s independent critical thinking; MW = meaningful work; WL = workload; ED = emotional dissonance.

Table 3 shows the results of the hierarchical multiple regression. As can be seen, JS was explained by six variables. Four were positively related—MW (β +.34, *p* < .001), F.AE (β +.13, *p* < .05), F.AE_squared (β +.10, *p* < .01) and tenure in organization (β +.10, *p* < .05) in decreasing order—and two, ED (β -.22, *p* < .001) and WL (β -.12, *p* < .05), were negatively related. The resulting R-square for the third model was 0.25 with an adjusted R-square of 0.23.

**Table 3:** Multiple regression results examining the linear and curvilinear effects of followership behaviors on JS.

		JS		
		Model 1	Model 2	Model 3
		*β*	*Β*	*β*
1.	Gender (1 = male)	.07	.07	.07
	Tenure in organization	.05	.10*	.10*
	Hours worked per week	.02	.06	.06
2.	F.AE		.10*	.13*
	F.ICT		-.03	-.05
	MW		.34**	.34**
	WL		-.12*	-.12*
	ED		-.21**	-.22**
3.	F.AE_squared			.10*
	F.ICT_squared			-.05
	*R*^*2*^	.006	.240	.250
	*Adjusted R*^*2*^	-.002	.224	.230
	*ΔR*^*2*^	.006	.234	.010

Note.

* *p*< .05;

** *p*< .001.

JS = job satisfaction; F.AE = follower’s active engagement; F.ICT = follower’s independent critical thinking; MW = meaningful work; WL = workload; ED = emotional dissonance.

Summarizing, the findings show that *Hp*. *1* was partially supported: there is a significant positive linear relationship between F.AE and JS but not between F.ICT and JS, and the first relationship is thus much stronger than the second. In other words, the more followers are engaged, the more they are satisfied with their job, while their thinking style does not have an impact on JS.

*Hp*. *2* received full support: there is also a U-shaped curvilinear relationship between F.AE and JS, after controlling for the linear relationship between the two variables. Respondents’ JS increases more than expected in a linear relationship when the level of engagement as a follower is very high.

*Hp*. *3* was not supported, since there is no curvilinear relationship between F.ICT and JS. As for the linear relationship, F.ICT does not show a significant relationship with JS. Thus followers’ thinking style seems to be irrelevant for their own satisfaction at work. It could also be, as mentioned earlier, that the link between these two variables is controversial and strongly moderated by the quality of the relationship with the leader (as for voice behaviors, see [33]). In other words, the relationship between F.ICT and JS could be significant and positive for followers who get along very well with their leader, and negative for followers who have a bad relationship.

*Hp*. *4* was supported and MW shows a significant positive linear relationship with JS. As the perception of respondents’ own job as meaningful increases, the perception of JS also increases: the more respondents think that their job is meaningful, the more they are satisfied with it.

*Hp*. *5* was fully supported since both ED and WL have a significant negative linear relationship with JS. The two job demands investigated in the study show an inverse relationship with JS: the higher ED and WL are for respondents the lower their JS was found to be.

## Discussion

### Theoretical implications

Summarizing the study findings, F.AE acts as a real resource for respondents’ JS, and it also shows a curvilinear relationship with the outcome variable. On the contrary, F.ICT did not show significant linear or curvilinear relationships with JS. Thus, it seems to be germane to the study of leadership dynamics investigating both followership behaviors and curvilinear relationships. The last resource investigated, MW, showed a strong impact on JS, as well as the two demands analyzed, WL and ED. These findings pinpoint a number of variables which seem crucial for the nursing profession.

Studying followership is particularly complex, since “a followership role is often ambiguous and open to interpretation” [68]. For this reason, the unconfirmed hypotheses about F.ICT (part of *Hp*. *1* and *Hp*. *3*) neither surprise nor discourage. But the inconsistent findings for this dimension obtained here as well as in previous studies [23, 24] call for rethinking its meaning and value. If we consider F.ICT as a proactive behavior like organizational citizenship behaviors or voice, where the latter can be regarded as the behavior closest to that of our followers, future studies could test an inverse relationship from satisfaction to F.ICT: JS could increase F.ICT as it does for other proactive behaviors [69]. In addition, it would be useful to test the moderating effect that LMX or a variable like the satisfaction for the leader has on the relationship between F.ICT and JS: if we reckon F.ICT as similar to voice behaviors we could expect that the outcome of that behavior is strongly influenced by the quality of the relationship with the leader (see [33]).

Our work follows one of the two promising theoretical framework for followership identified by Uhl-Bien and colleagues [17]; the role-based approach, which sets out to “reverse the lens” [70] and concentrate on followers. This study demonstrates that followership behaviors have a significant impact on followers’ well-being, and particularly on their JS. This outcome, and more in general this perspective, was not been directly examined by the authors of the 2014 [17] review (who instead stress the investigation of leaders’ outcomes linked to their followers’ behavior, or “the leader side” of the leadership story, as the authors call it). However, we consider this perspective particularly interesting, in view of the number of followers that work in every organization [3, 71] and their importance for achieving organizational goals [3, 4].

JS has received significant attention in the past, and so it is difficult to add something new to the hundreds of studies on its determinants. First of all, in fact, the study confirmed the value of some classical demands and resources as JS antecedents: WL, ED, and MW have a significant relationship with the investigated outcome. The latter variable in particular has a strong impact on JS: this was expected from the JD-R theory, which hypothesizes that resources have a stronger impact than demands on motivational and positive outcomes, and from the job characteristic model by Hackman and Oldham [51], who emphasized the close link between the psychological state of meaningfulness of the work and general JS [52]. This “traditional” link has re-gained prominence in today’s labor market, where the psychological contract with the organization [72] and the external and extrinsic sources of motivation are becoming weaker and weaker for many employees, especially those in lower positions. In addition, again taking into consideration the characteristics of the labor market and the changes in the nursing profession, it is interesting to investigate the relationship between MW and JS in this sample. In a certain sense, finding such a high beta coefficient in this multiple regression, which investigates many antecedents, is a positive result which also gives information about how the majority of nurses still interpret their profession. Though the antecedents have the expected impacts on the outcome variable, the same cannot be said for the control variables: JS has no significant correlations with them, even if the literature shows some association between JS and gender (e.g., [73–75]), JS and tenure in the organization (e.g., [76]), JS and hours worked per week [77, 78].

In any case, this study’s most salient finding is that F.AE has a linear and curvilinear impact on JS. The relationship that people have with their leader can increase their JS when they think about themselves not only as passive recipients of that relationship, but also as active interpreters of their followers’ role. Being an active engaged follower is positive and it seems even more positive when engagement reaches a very high level.

From a theoretical standpoint, this also emphasizes the value of investigating curvilinear relationships between well-being variables and their antecedents. It would be worthwhile to summarize the literature on well-being at work which suggests analyzing curvilinear relationships, pinpointing its potential inconsistencies and stable findings. Thinking in particular of resources, this would call for cataloguing and reflecting on the characteristics that resources need in order to show positive curvilinear relationships, such as the one we found here, or negative curvilinear relationships on well-being outcomes. It would be very important to identify those resources that have a positive exponential impact on well-being outcomes and those that, after a certain level, interrupt their positive impact or even start being harmful (as is hypothesized in the vitamin model, see for instance [26] for a detailed description of the model).

### Practical implications

It is well-known that more satisfied employees are also more motivated and show higher performance [79], and that they have lower turnover intentions [41, 79]. Likewise, meta-analytical studies have demonstrated that, for nurses, JS has a strong inverse association with stress [37, 38] and a strong positive association with organizational commitment [37] and nurse–physician collaboration [38]. These relationships emphasize the practical value of this study.

Specifically, some practical implications follow from the role of F.AE on JS. Fostering followers’ proactivity, recognizing and supporting it, for instance through training programs which raise supervisors’ awareness and develop leaders who are open to proactivity, would be important, particularly in view of the resistance and negative stereotypes that have been observed for supervisees’ proactive behaviors [80]. Taking measures which facilitate F.AE seems to have a very positive impact on their JS. This could be supported, for instance, by a transformational style of leadership, whose main aim is to transform followers into new leaders [81], or by a generative style of leadership, which, in the idea of “balance”, finds space for followers, for the relationship with them and for their initiative [82].

The very high impact of MW on JS emphasizes the importance of recognizing the value of people’s work, of reaffirming what a profession really means with appropriate organizational communication. In the case of the nursing profession which has a clear social value such recognition could be easier to convey. Carefully managed internal communication about the value of nurses, and which is targeted to all of the many professions that work in a hospital, increase nurses’ JS and well-being. In addition, a good communication strategy could partially address the emotional load involved in nursing, clarifying that it is part of the profession but that there are different strategies for coping with it. A more concerted effort to lessen ED could entail working on emotions in specific training programs with nurses or with even more individualized in-depth measures such as coaching and counseling [82].

### Limitations and future research

As for the study limitations, first of all the data were collected through a cross-sectional research design and a self-report questionnaire. Cross-sectional designs involve a risk of common method bias, which future studies could lessen, for instance by investigating leaders’ perceptions of followers’ behaviours in addition to followers’ self-perceptions. Using a self-report questionnaire can lead to problems with self-report bias, and particularly with social desirability, since previous studies showed a significant relationship between F.AE and F.ICT and a Lie scale [24]. A second limitation is linked to potential influences on followers’ behaviors which were not taken into consideration in the study but can change followers’ perception of these behaviors. In responding to questions that use behavioral scales to measure leadership styles, followers combine their perceptions of leadership with other aspects of the situation [83]. In addition, measures of leadership styles have a structure that emphasizes behavior but, at a deeper level, they reflect both the social and personal context within which measurement occurred [84]. Consequently, in measuring followers’ behaviors, we could at the same time be measuring context characteristics which encourage these behaviors, making them egosyntonic and so capable of increasing JS and well-being. The limitation is thus that we did not consider these contextual elements which can influence the analyzed behaviors, and that we did not consider the followers’ evaluations of these behaviors. In fact, as Carsten and colleagues mention [5], whether followers are able to act consistently with their schema or preferences hinges on the context. “When followers’ schemas do not match the context, they report stress and dissatisfaction. For example, […] proactive followers with authoritarian leaders report frustration and dissatisfaction from being stifled by bureaucratic climates and procedures” ([17], p. 91). A final limitation is that the findings of this study are not generalizable to Italian nurses, even though Italian hospitals are fairly similar in terms of structures and processes and even though the respondents’ distribution is in line with national data and other Italian studies as regards gender. Specifically, according to data from the Italian nurses’ association IPASVI [85], 77.3% of the nursing population in 2015 consisted of women, and in a recently published study on a different Italian hospital [86], 86% of respondents were females, while 86.6% of respondents in our sample are females. To test generalizability, it will be necessary to replicate this study in hospitals which are located in other parts of Italy and which differ in size (the hospital investigated in this study is located in North-West Italy and is of medium size). Lastly, although these findings are not generalizable, there are some points in common with other studies carried out in different countries which used large samples: for instance, on a representative sample of 1141 registered nurses in 34 US states, researchers found that quantitative workload explains job satisfaction in a model that tests several other predictors [87]; again on a sample of registered nurses in the US, the “cover” sub-strategy of the behavior called surface acting—which strictly recalls emotional dissonance—decreased women’s job satisfaction after controlling for several variables [88]; on a sample of 3.088 female nurses in China, researchers found a strong positive correlation between job satisfaction and meaning of work [89].

Future research could adopt a dyadic approach to also investigate supervisors’ leadership style and their evaluation of followers’ proactivity. Analyzing the supervisors’ leadership style could be valuable in order to measure one of the contextual variables that can influence followers’ behaviors as discussed above. Analyzing supervisors’ evaluation of proactive behaviors would be of great interest, particularly in view of what Grant and colleagues wrote about this topic ([80], p. 32): “supervisors do not always appreciate proactivity. Researchers have begun to point out that supervisors may see proactive behavior as a threat (Frese & Fay, 2001; Miceli & Near, 1994; Parker et al., 2006), an ingratiation attempt (Bolino, 1999), or an ill-timed distraction (Chan, 2006)”. Not being open to followers’ proactivity certainly has a strong effect on their concrete behavior, and it could be important to gauge its impact in order to collect findings that can be used in training and communication.

## Supporting information

S1 Appendix Abbreviations for the study variables. (DOCX)

S1 Questionnaire (English version). Satisfaction and work relationships.
(DOCX)

S2 Questionnaire (Italian version). Soddisfazione e relazioni di lavoro.
(DOCX)

S1 Data matrix (SPSS). The data matrix with all the information for replicating the study.
(SAV)

## References

[1] Kelley RE. The Power of Followership: How to create leaders people want to follow, and followers who lead themselves. New York: Doubleday; 1992.

[2] Agho AO. Perspectives of senior-level executives on effective followership and leadership. Journal of Leadership & Organizational Studies. 2009; 16(2): 159–166.

[3] Collinson D. Rethinking followership: A post–structuralist analysis of follower identities. Leadersh Q. 2006; 17(2): 179–189.

[4] Kelley RE. In praise of followers. Harv Bus Rev. 1988; 66(6): 142–148.

[5] Carsten MK, Uhl-Bien M, West BJ, Patera JL, McGregor R. Exploring social constructions of followership: A qualitative study. Leadersh Q. 2010; 21(3): 543–562.

[6] Dulebohn JH, Bommer WH, Liden RC, Brouer RL, Ferris GR. A meta-analysis of antecedents and consequences of leader-member exchange integrating the past with an eye toward the future. J Manage. 2012; 38(6): 1715–1759.

[7] Demerouti E, Bakker AB, Nachreiner F, Schaufeli WB. The job demands-resources model of burnout. J Appl Psychol. 2001; 86(3): 499–512. 11419809

[8] Zito M, Cortese CG, Colombo L. Nurses’ exhaustion: the role of flow at work between job demands and job resources. J Nurs Manag. 2016; 24(1): 1–11. 25612156 10.1111/jonm.12284

[9] Bakker AB, Oerlemans W. Subjective well-being in organizations In: Cameron KS, Spreitzer GM, editors. The Oxford Handbook of Positive Organizational Scholarship. New York: Oxford University Press; 2011 pp. 178–189.

[10] Kirpal S. Work identities of nurses: Between caring and efficiency demands. Career Development International. 2004; 9(3): 274–304.

[11] Schulter PJ, Turner C, Huntington AD, Bain CJ, McClure RJ. Work/life balance and health: The nurses and midwives e-cohort study. Int Nurs Rev. 2011; 58(1): 28–36. doi: 10.1111/j.1466-7657.2010.00849.x 21281290 10.1111/j.1466-7657.2010.00849.x

[12] Bakker AB, Demerouti E. The job demands-resources model: State of the art. Journal of Managerial Psychology. 2007; 22(3): 309–328.

[13] Demerouti E, Bakker AB. The Job Demands-Resources model: Challenges for future research. South African Journal of Industrial Psychology. 2011; 37: 1–9.

[14] Schaufeli WB, Taris TW. A critical review of the Job Demands-Resources Model: Implications for improving work and health In: Bauer GF, Hämmig F, editors. Bridging occupational, organizational and public health. Netherlands: Springer; 2014 pp. 43–68.

[15] Harris KJ, Kacmar KM. Too much of a good thing: The curvilinear effect of leader-member exchange on stress. J Soc Psychol. 2006; 146(1): 65–84. doi: 10.3200/SOCP.146.1.65-84 16480122 10.3200/SOCP.146.1.65-84

[16] Hyrkas K. Editorial. Management and leadership at the bedside. J Nurs Manag. 2012; 20: 579–581. doi: 10.1111/j.1365-2834.2012.01476.x 22823212 10.1111/j.1365-2834.2012.01476.x

[17] Uhl-Bien M, Riggio RE, Lowe KB, Carsten MK. Followership theory: A review and research agenda. Leadersh Q. 2014; 25(1): 83–104.

[18] Kelley RE. Rethinking followership In: Riggio R, Chaleff I, Lipman-Blumen J, editors. The art of followership: How great followers create great leaders and organizations. San Francisco: Jossey-Bass; 2008 pp. 5–16.

[19] Morrison EW, Employee voice behavior: Integration and directions for future research. Acad Manage A. 2011; 5(1), 373–412.

[20] Tanoff GF, Barlow C. Leadership and followership: Same animal, different spots? Consulting Psychology Journal: Practice and Research. 2002; 54(3): 157–167.

[21] Mushonga S, Torrance C. Assessing the relationship between followership and the big five factor model of personality. Review of Business Research. 2008; 8(2): 185–193.

[22] Gatti P, Cortese CG, Tartari M, Ghislieri C. Followers’ active engagement: Between personal and organizational dimensions. BPA-Applied Psychology Bulletin (Bollettino di Psicologia Applicata). 2014; 62(270): 2–11.

[23] Blanchard AL, Welbourne J, Gilmore D, Bullock A. Followership styles and employee attachment to organization. The Psychologist-Manager Journal. 2009; 12(2): 111–131.

[24] Gatti P, Tartari M, Cortese CG, Ghislieri C. A contribution to the Italian validation of Kelley’s followership questionnaire. TPM Test Psychom Methodol Appl Psychol. 2014; 21(1): 67–87.

[25] Janssen O. Fairness perceptions as a moderator in the curvilinear relationships between job demands, and job performance and job satisfaction. Acad Manage J. 2001; 44(5): 1039–1050.

[26] Warr P. Work, unemployment, and mental health. Oxford: Oxford University Press; 1987.

[27] Hochwarter W, Byrne ZS. LMX and job tension: Linear and non-linear effects and affectivity. J Bus Psychol. 2005; 19(4): 505–520.

[28] Hesselgreaves H, Scholarios D. Leader–member exchange and strain: A study of job demands and role status. Hum Resour Manage. 2014; 24(4): 459–478.

[29] Chung-Yan GA, Butler AM. Proactive personality in the context of job complexity. Can J Behav Sci. 2011; 43(4): 279–286.

[30] Li N, Liang J, Crant JM. The role of proactive personality in job satisfaction and organizational citizenship behavior: A relational perspective. J Appl Psychol. 2010; 95(2): 395–404. doi: 10.1037/a0018079 20230079 10.1037/a0018079

[31] Van Dyne L, LePine JA. Helping and extra-role behavior: Evidence of construct and predictive validity. Acad Manage J. 1998; 41(1): 108–119.

[32] LePine JA, Van Dyne L. Voice and cooperative behavior as contrasting forms of contextual performance: Evidence of differential relationships with big five personality characteristics and cognitive ability. J Appl Psychol. 2001; 86(2): 326–336. 11393444 10.1037/0021-9010.86.2.326

[33] Detert JR, Burris ER. Leadership behavior and employee voice: Is the door really open?. Acad Manage J. 2007; 50(4): 869–884.

[34] Tangirala S, Ramanujam R. Exploring nonlinearity in employee voice: The effects of personal control and organizational identification. Acad Manage J. 2008; 51(6): 1189–1203.

[35] Qin X, DiRenzo MS, Xu M, Duan Y. When do emotionally exhausted employees speak up? Exploring the potential curvilinear relationship between emotional exhaustion and voice. J Organ Behav. 2014; 35(7): 1018–1041.

[36] Locke EA. The nature and causes of job satisfaction In: Dunnette M, editor. Handbook of Industrial and Organizational Psychology. Chicago: Rand McNally; 1976 pp. 1297–1350.

[37] Blegen MA. Nurses’ job satisfaction: A meta-analysis of related variables. Nurs Res. 1993; 42(1): 36–41. 8424066

[38] Zangaro GA, Soeken KL. A meta-analysis of studies of nurses’ job satisfaction. Res Nurs Health. 2007; 30(4): 445–458. doi: 10.1002/nur.20202 17654483 10.1002/nur.20202

[39] Cortese CG. Job satisfaction of Italian nurses: An exploratory study. J Nurs Manag. 2007; 15(3): 303–312. doi: 10.1111/j.1365-2834.2007.00694.x 17359430 10.1111/j.1365-2834.2007.00694.x

[40] Cortese CG. Job satisfaction among nursing personnel: Application of the Italian version of the Stamps Index of Work Satisfaction (1997). Med Lav. 2007; 98(3): 175–191. Italian. 17598346

[41] Cortese CG. Predictors of intention to leave the nursing profession in two Italian hospitals. Assist Inferm Ric. 2013; 32(1): 20–27. Italian. doi: 10.1702/1267.13987 23644759 10.1702/1267.13987

[42] Mauno S, Kinnunen U, Ruokolainen M. Exploring work-and organization-based resources as moderators between work–family conflict, well-being, and job attitudes. Work Stress. 2006; 20(3): 210–233.

[43] Nielsen MB, Mearns K, Matthiesen SB, Eid J. Using the Job Demands-Resources model to investigate risk perception, safety climate and job satisfaction in safety critical organizations. Scand J Psychol. 2011; 52(5): 465–475. doi: 10.1111/j.1467-9450.2011.00885.x 21534979 10.1111/j.1467-9450.2011.00885.x

[44] Hall GB, Dollard MF, Winefield AH, Dormann C, Bakker AB. Psychosocial safety climate buffers effects of job demands on depression and positive organizational behaviors. Anxiety Stress Coping. 2013; 26(4): 355–377. doi: 10.1080/10615806.2012.700477 22793792 10.1080/10615806.2012.700477

[45] Bradley JR, Cartwright S. Social support, job stress, health, and job satisfaction among nurses in the United Kingdom. Int J Stress Manag. 2002; 9(3): 163–182.

[46] Han K, Trinkoff AM, Gurses AP. Work-related factors, job satisfaction and intent to leave the current job among United States nurses. J Clin Nurs. 2015; 24(21–22): 3224–3232. doi: 10.1111/jocn.12987 26417730 10.1111/jocn.12987

[47] Lieff SJ. Perspective: The missing link in academic career planning and development: pursuit of meaningful and aligned work. Acad Med. 2009; 84(10): 1383–1388. doi: 10.1097/ACM.0b013e3181b6bd54 19881426 10.1097/ACM.0b013e3181b6bd54

[48] Arnold KA, Turner N, Barling J, Kelloway EK, McKee MC. Transformational leadership and psychological well-being: The mediating role of meaningful work. J Occup Health Psychol. 2007; 12(3): 193–203. doi: 10.1037/1076-8998.12.3.193 17638487 10.1037/1076-8998.12.3.193

[49] Kazemipour F, Mohd Amin S. The impact of workplace spirituality dimensions on organisational citizenship behaviour among nurses with the mediating effect of affective organisational commitment. J Nurs Manag. 2012; 20(8): 1039–1048. doi: 10.1111/jonm.12025 23151106 10.1111/jonm.12025

[50] Cortese CG, Gatti P, Ghislieri C. Job demands, meaningful work, and turnover intention among nurses. Med Lav. 2014; 105(1): 37–47. Italian. 24552093

[51] Hackman JR, Oldham GR. Motivation through the design of work: Test of a theory. Organ Behav Hum Perform. 1976; 16: 250–279.

[52] Fried Y, Ferris GR. The validity of the job characteristics model: A review and meta-analysis. Pers Psychol. 1987; 40: 287–322.

[53] Jönsson S. Psychosocial work environment and prediction of job satisfaction among Swedish registered nurses and physicians–a follow-up study. Scand J Caring Sci. 2012; 26(2): 236–244. doi: 10.1111/j.1471-6712.2011.00924.x 21923675 10.1111/j.1471-6712.2011.00924.x

[54] Atefi N, Abdullah KL, Wong LP, Mazlom R. Factors influencing registered nurses perception of their overall job satisfaction: A qualitative study. Int Nurs Rev. 2014; 61(3): 352–360. doi: 10.1111/inr.12112 24902878 10.1111/inr.12112

[55] van Veldhoven MJ, Beijer SE. Workload, work-to-family conflict, and health: Gender differences and the influence of private life context. J Soc Issues. 2012; 68(4): 665–683.

[56] Zeytinoglu IU, Denton M, Davies S, Baumann A, Blythe J, Boos L. Deteriorated external work environment, heavy workload and nurses’ job satisfaction and turnover intention. Can Public Policy. 2007; 33(Supplement 1): S31–S47.

[57] Stuart EH, Jarvis A, Daniel K. A ward without walls? District nurses’ perceptions of their workload management priorities and job satisfaction. J Clin Nurs. 2008; 17(22): 3012–3020. doi: 10.1111/j.1365-2702.2008.02316.x 18647197 10.1111/j.1365-2702.2008.02316.x

[58] Bakker AB, Heuven E. Emotional dissonance, burnout, and in-role performance among nurses and police officers. Int J Stress Manag. 2006; 13(4): 423–440.

[59] Zapf D, Vogt C, Seifert C, Mertini H, Isic A. Emotion work as a source of stress: The concept and development of an instrument. European Journal of Work and Organizational Psychology. 1999; 8: 371–400.

[60] Lewig KA, Dollard MF. Emotional dissonance, emotional exhaustion and job satisfaction in call centre workers. European Journal of Work and Organizational Psychology. 2003; 12: 366–392.

[61] World Medical Association. World Medical Association Declaration of Helsinki. Ethical principles for medical research involving human subjects. Bull World Health Organ, 2001; 79(4): 373–374. 11357217 PMC2566407

[62] Pejtersen JH, Kristensen TS, Borg V, Bjorner JB. The second version of the Copenhagen Psychosocial Questionnaire. Scand J Public Health Suppl. 2010; 8 (Suppl 3): 8–24. 10.1177/1403494809349858 21172767

[63] Ghislieri C, Gatti P, Cortese CG. A brief scale for investigating followership in nursing. BPA-Applied Psychology Bulletin (Bollettino di Psicologia Applicata). 2015; 63(272): 25–32.

[64] Leiter MP, Harvie P, Frizzell C. The correspondence of patient satisfaction and nurse burnout. Soc Sci Med. 1998; 47: 1611–1617. 9823056 10.1016/s0277-9536(98)00207-x

[65] Ashmos DP, Duchon D. Spirituality at work: A conceptualization and measurement. Journal of Management Inquiry. 2000; 9: 134–145.

[66] Bakker AB, Demerouti E, Verbeke W. Using the job demands-resources model to predict burnout and performance. Hum Resour Manage. 2004; 43: 83–104.

[67] Nunnally JC. Psychometric theory. 2nd ed New York: McGraw-Hill; 1978.

[68] Parker SK. ‘That is my job’: How employees role orientation affects their job performance. Human Relations. 2007; 60(3): 403–434.

[69] Williams LJ, Anderson SE. Job satisfaction and organizational commitment as predictors of organizational citizenship and in-role behaviors. J Manage. 1991; 17(3): 601–617.

[70] Shamir B. From passive recipients to active co-producers: Followers’ roles in the leadership process In: Shamir B, Pillai R, Bligh M, Uhl-Bien M, editors. Follower-centered perspectives on leadership: A tribute to the memory of James R. Meindl. Charlotte: Information Age Publishing; 2007 pp. 9–39.

[71] Steger JA, Manners GE Jr, Zimmerer TW. Following the leader: How to link management style to subordinate personalities. Manage Rev. 1982; 71(10): 22–51. 10262397

[72] Robinson SL, Rousseau DM. Violating the psychological contract: Not the exception but the norm. J Organ Behav. 1994; 15: 245–259.

[73] Clark AE. Job satisfaction and gender: Why are women so happy at work?. Labour Econ. 1997; 4(4): 341–372.

[74] Magee W. Anxiety, demoralization, and the gender difference in job satisfaction. Sex Roles. 2013; 69(5–6): 308–322.

[75] Singhapakdi A, Sirgy MJ, Lee DJ, Senasu K, Grace BY, Nisius AM. Gender disparity in job satisfaction of Western versus Asian managers. J Bus Res. 2014; 67(6): 1257–1266.

[76] Katz R. Job longevity as a situational factor in job satisfaction. Adm Sci Q. 1978; 204–223. 10307891

[77] Hills D, Joyce C, Humphreys J. Validation of a job satisfaction scale in the Australian clinical medical workforce. Eval Health Prof. 2011; 35(1): 47–76. doi: 10.1177/0163278710397339 21411473 10.1177/0163278710397339

[78] Joyce C, Wang WC. Job satisfaction among Australian doctors: The use of latent class analysis. J Health Serv Res Policy. 2015; 20(4): 224–230. doi: 10.1177/1355819615591022 26079142 10.1177/1355819615591022

[79] Kinicki AJ, McKee-Ryan FM, Schriesheim CA, Carson KP. Assessing the construct validity of the job descriptive index: A review and meta-analysis. J Appl Psychol. 2002; 87(1): 14–32. 11916208 10.1037/0021-9010.87.1.14

[80] Grant AM, Parker S, Collins C. Getting credit for proactive behavior: Supervisor reactions depend on what you value and how you feel. Pers Psychol. 2009; 62(1): 31–55.

[81] Burns JM. Leadership. New York: Harper Collins; 1978.

[82] Ghislieri C, Gatti P. Generativity and balance in leadership. Leadership, 2012; 8(3): 257–275.

[83] Hansbrough TK, Lord RG, Schyns B. Reconsidering the accuracy of follower leadership ratings. Leadersh Q, 2015; 26(2): 220–237.

[84] Lord RG, Gatti P, Chui SL. Social-cognitive, relational, and identity-based approaches to leadership. Organ Behav Hum Decis Process. 2016; 136: 119–134.

[85] IPASVI—Federazione Nazionale Collegi Infermieri [Internet]. Professione Infermiere. 2015 [cited 2017 May 23]. www.ipasvi.it/archivio_news/pagine/256/8_scheda_Professione_Infermiere.pdf

[86] Ghislieri C, Gatti P, Molino M, Cortese CG. Work–family conflict and enrichment in nurses: between job demands, perceived organisational support and work–family backlash. J Nurs Manag. 2017; 25(1): 65–75. doi: 10.1111/jonm.12442 27859839 10.1111/jonm.12442

[87] Djukic M, Kovner CT, Brewer CS, Fatehi F, Greene WH. Exploring direct and indirect influences of physical work environment on job satisfaction for early-career registered nurses employed in hospitals. Res Nurs Health. 2014; 37(4): 312–325. doi: 10.1002/nur.21606 24985551 10.1002/nur.21606

[88] Cottingham MD, Erickson RJ, Diefendorff JM. Examining men’s status shield and status bonus: How gender frames the emotional labor and job satisfaction of nurses. Sex Roles. 2015; 72(7–8): 377–389.

[89] Li J, Fu H, Hu Y, Shang L, Wu Y, Kristensen TS, et al Psychosocial work environment and intention to leave the nursing profession: Results from the longitudinal Chinese NEXT study. Scand J Public Health Suppl. 2010; 38(3): 69–80. 10.1177/1403494809354361 21172773

