# Supplementary material for: Relationships between followers’ behaviors and job satisfaction in a sample of nurses
Source: PLoS One. 2017 Oct 5;12(10):e0185905. doi: 10.1371/journal.pone.0185905 (PMC5628884; doi:10.1371/journal.pone.0185905)
Supplement: S1 Appendix — (DOCX) [file pone.0185905.s001.docx]

**Appendix**

**Abbreviations for the study variables (in alphabetical order)**

| Abbreviation | Variable name |
| --- | --- |
| ED | Emotional dissonance |
| F.AE | Follower’s active engagement |
| F.ICT | Follower’s independent critical thinking |
| JS | Job satisfaction |
| MW | Meaningful work |
| WL | Workload |
