# Supplementary material for: Relationships between followers’ behaviors and job satisfaction in a sample of nurses
Source: PLoS One. 2017 Oct 5;12(10):e0185905. doi: 10.1371/journal.pone.0185905 (PMC5628884; doi:10.1371/journal.pone.0185905)
Supplement: S1 Questionnaire — Satisfaction and work relationships. (DOCX) [file pone.0185905.s002.docx]

**QUESTIONNAIRE – SATISFACTION AND WORK RELATIONSHIPS**

1. *SECTION A – SOCIO-DEMOGRAPHIC CHARACTERISTICS*

#### 1. Gender

| 🞎 1 | Female |
| --- | --- |
| 🞎 2 | Male |

**2. Age:___**________years

**3. Educational level**

| 🞎 1 | Elementary school |
| --- | --- |
| 🞎 2 | Middle school |
| 🞎 3 | High school diploma |
| 🞎 4 | Bachelor’s degree |
| 🞎 5 | Master’s degree |
| 🞎 6 | PhD or similar |
| 🞎 7 | Other (*specify*): |

**4. What type of contract do you have?**

| 🞎 1 | Permanent contract |
| --- | --- |
| 🞎 2 | Temporary contract |

**5. What is your work schedule?**

| 🞎 1 | Full-time |
| --- | --- |
| 🞎 2 | Part-time |

**6. How long have you worked in your current organization?**

**______________years**

**7. In your entire career, how long have you worked?**

**______________years**

**8. How many hours do you work per week (on average)? ______________hours**

**9. What area do you work in?**

| ** 1** | **Medicine** |
| --- | --- |
| ** 2** | **Surgery** |
| ** 3** | **Intensive care** |
| ** 4** | **Service area** |

1. *SECTION B – INDIVIDUAL-ORGANIZATION INTERACTION AND JOB CHARACTERISTICS*

**10. The following questions regard your situation on the job and how you perceive it. Please indicate, how often…**

|  |  | 1  Never | 5  Always |
| --- | --- | --- | --- |
| 1 | Do you have to work at speed?* | ① ② ③ ④ ⑤ | |
| 2 | Do you have too much work to do? | ① ② ③ ④ ⑤ | |
| 3 | How often do you have to work extra hard in order to reach a deadline? | ① ② ③ ④ ⑤ | |
| 4 | Do you work under time pressure? | ① ② ③ ④ ⑤ | |

* In the Italian version we put statements rather than questions, as in the original items, because it sounded clearer to us. In any case, the meaning of the items does not change.

**11. Thinking about your job in general, please state your level of agreement with the following statements.**

|  |  | 1  Strongly disagree | 7  Strongly  agree |
| --- | --- | --- | --- |
| 1 | The work I do is connected to what I think is important in life | ① ② ③ ④ ⑤ ⑥ ⑦ | |
| 2 | I see a connection between my work and the larger social good of my community | ① ② ③ ④ ⑤ ⑥ ⑦ | |
|  |  | 1  Strongly disagree | 7  Strongly  agree |
| 3 | This job provides me with opportunities to do work which I feel is important | ① ② ③ ④ ⑤ ⑥ ⑦ | |
| 4 | I have clear personal goals I wish to accomplish through doing this job well | ① ② ③ ④ ⑤ ⑥ ⑦ | |
| 5 | My job provides me with successes which make me feel great | ① ② ③ ④ ⑤ ⑥ ⑦ | |

12. Thinking of your working day, please state how often you have to….

|  |  | 1  Never | 6  Always |
| --- | --- | --- | --- |
| 1 | Display emotions which do not correspond to inner feelings | ① ② ③ ④ ⑤ ⑥ | |
| 2 | Display positive emotions while feeling indifferent | ① ② ③ ④ ⑤ ⑥ | |
| 3 | Force yourself to show certain feelings | ① ② ③ ④ ⑤ ⑥ | |

**13. Thinking of your job in general, how satisfied are you with the following aspects?**

|  |  | 1  Very dissatisfied | 5  Very satisfied |
| --- | --- | --- | --- |
| 1 | …your work prospects?* | ① ② ③ ④ ⑤ | |
| 2 | …the physical working conditions? | ① ② ③ ④ ⑤ | |
| 3 | …the way your abilities are used? | ① ② ③ ④ ⑤ | |
| 4 | …your job as a whole, everything taken into consideration? | ① ② ③ ④ ⑤ | |

* In the Italian version we put statements rather than questions, as in the original items, because it sounded clearer to us. In any case, the meaning of the items does not change.

**SECTION C – THE RELATIONSHIP WITH YOUR BOSS/DIRECT SUPERVISOR**

The following questions regard your direct supervisor and you as his/her supervisee/follower:

**NB. DIRECT SUPERVISOR = your head nurse, not the physician who heads the ward where you work.**

**14. For each question, please indicate how much it applies to you, thinking of specific situations in which you work as the supervisee/follower of your boss/direct supervisor.**

|  |  | 0  Never | 6  Always |  |
| --- | --- | --- | --- | --- |
| 1 | When starting a new assignment, do you promptly build a record of successes in tasks that are important to your departmental chairperson*? | ⓪ ① ② ③ ④ ⑤ ⑥ | |  |
| 2 | Do you take the initiative to seek out and successfully complete assignments that go above and beyond your job? | ⓪ ① ② ③ ④ ⑤ ⑥ | |  |
| 3 | Do you independently think up and champion new ideas that will contribute significantly to your departmental chairperson’s or your department’s goals? | ⓪ ① ② ③ ④ ⑤ ⑥ | | |
| 4 | Do you try to solve the tough problems (technical or organizational), rather than look to your departmental chairperson to do it for you? | ⓪ ① ② ③ ④ ⑤ ⑥ | |  |
| 5 | Do you make a habit of internally questioning the wisdom of your departmental chairperson’s decision rather than just doing what you are told? | ⓪ ① ② ③ ④ ⑤ ⑥ | |  |
| 6 | When your departmental chairperson asks you to do something that runs contrary to your professional or personal preferences, do you say ‘no’ rather than ‘yes’? | ⓪ ① ② ③ ④ ⑤ ⑥ | |  |
| 7 | Do you act on your own ethical standards rather than your departmental chairperson’s or your department’s standards? | ⓪ ① ② ③ ④ ⑤ ⑥ | |  |
|  |  | 0  Never | 6  Always |  |
| 8 | Do you assert your views on important issues, even though it might mean conflict with your group or reprisals from your departmental chairperson? | ⓪ ① ② ③ ④ ⑤ ⑥ | |  |

* “Departmental chairperson” is the expression used in the original version of the scale. In the Italian version we put a word that usually people use in ordinary speech that could be translated in English as “boss” or “direct supervisor”.

**Thanks a lot for the time you devoted to filling in the questionnaire!**
