# Supplementary material for: Relationships between followers’ behaviors and job satisfaction in a sample of nurses
Source: PLoS One. 2017 Oct 5;12(10):e0185905. doi: 10.1371/journal.pone.0185905 (PMC5628884; doi:10.1371/journal.pone.0185905)
Supplement: S2 Questionnaire — Soddisfazione e relazioni di lavoro. (DOCX) [file pone.0185905.s003.docx]

**QUESTIONARIO – SODDISFAZIONE E RELAZIONI DI LAVORO**

1. *SEZIONE A – CARATTERISTICHE SOCIO-DEMOGRAFICHE*

#### 1. Genere

| 🞎 1 | Donna |
| --- | --- |
| 🞎 2 | Uomo |

**2. Età:___**________anni

**3. Titolo di studio**

| 🞎 1 | Licenza di scuola elementare |
| --- | --- |
| 🞎 2 | Diploma di scuola media inferiore |
| 🞎 3 | Diploma di scuola media superiore |
| 🞎 4 | Diploma universitario/Laurea triennale |
| 🞎 5 | Laurea vecchio ordinamento/Laurea specialistica |
| 🞎 6 | Specializzazione universitaria post-laurea |
| 🞎 7 | Altro (*specificare*): |

**4. Qual è il suo tipo di contratto?**

| 🞎 1 | A tempo indeterminato |
| --- | --- |
| 🞎 2 | A tempo determinato |

**5. Qual è il suo regime orario?**

| 🞎 1 | Full-time |
| --- | --- |
| 🞎 2 | Part-time |

**6. Da quanto tempo lavora in questa azienda?**

**______________anni**

**7. Qual è la sua anzianità lavorativa complessiva?**

**______________anni**

**8. Quante ore lavora in media la settimana?______________ore**

**9. Indichi quale è la sua area lavorativa di appartenenza:**

| ** 1** | **Medicina** |
| --- | --- |
| ** 2** | **Chirurgia** |
| ** 3** | **Area critica** |
| ** 4** | **Servizi** |

1. *SEZIONE B – INTERAZIONE SOGGETTO-ORGANIZZAZIONE E CRATTERISTICHE DEL LAVORO*

**10. Le seguenti affermazioni fanno riferimento alla sua personale situazione di lavoro e a come lei ne fa esperienza. Le chiediamo di indicare con quale frequenza...**

|  |  | 1  Mai | 5  Sempre |
| --- | --- | --- | --- |
| 1 | Deve lavorare velocemente | ① ② ③ ④ ⑤ | |
| 2 | Ha troppo lavoro da fare | ① ② ③ ④ ⑤ | |
| 3 | Deve lavorare più intensamente del solito per rispettare una scadenza | ① ② ③ ④ ⑤ | |
| 4 | Lavora sotto pressione | ① ② ③ ④ ⑤ | |

**11. Pensando al suo lavoro in generale, le chiediamo di indicare il suo grado di accordo con le seguenti affermazioni.**

|  |  | 1  Fortemente in disaccordo | 7  Fortemente d'accordo |
| --- | --- | --- | --- |
| 1 | Il lavoro che faccio è collegato con ciò che penso sia importante nella vita | ① ② ③ ④ ⑤ ⑥ ⑦ | |
| 2 | Vedo un legame tra il mio lavoro e il bene della mia comunità | ① ② ③ ④ ⑤ ⑥ ⑦ | |

|  |  | 1  Fortemente in disaccordo | 7  Fortemente d'accordo |
| --- | --- | --- | --- |
| 3 | Questa professione mi dà l’opportunità di fare un lavoro che sento come importante | ① ② ③ ④ ⑤ ⑥ ⑦ | |
| 4 | Ho dei chiari obiettivi personali che desidero raggiungere facendo bene questo lavoro | ① ② ③ ④ ⑤ ⑥ ⑦ | |
| 5 | Il mio lavoro mi permette di ottenere successi che mi fanno sentire molto bene | ① ② ③ ④ ⑤ ⑥ ⑦ | |

12. Pensando alla sua giornata lavorativa, indichi con quale frequenza le è richiesto di…

|  |  | 1  Mai | 6  Sempre |
| --- | --- | --- | --- |
| 1 | ...mostrare emozioni che non corrispondono ai suoi reali sentimenti | ① ② ③ ④ ⑤ ⑥ | |
| 2 | ...mostrare emozioni positive anche se si sente indifferente | ① ② ③ ④ ⑤ ⑥ | |
| 3 | ...sforzarsi di mostrare determinati sentimenti | ① ② ③ ④ ⑤ ⑥ | |

**13. Pensando al suo lavoro in generale, le chiediamo di indicare la sua soddisfazione rispetto ai seguenti aspetti:**

|  |  | 1  Molto  insoddisfatto | 5  Molto  soddisfatto |
| --- | --- | --- | --- |
| 1 | Le sue prospettive di lavoro | ① ② ③ ④ ⑤ | |
| 2 | Le condizioni fisiche di lavoro | ① ② ③ ④ ⑤ | |
| 3 | Il modo in cui vengono utilizzate le sue capacità | ① ② ③ ④ ⑤ | |
| 4 | Il suo lavoro nel complesso, tenendo in considerazione ogni elemento | ① ② ③ ④ ⑤ | |

*SEZIONE C – LA RELAZIONE CON IL CAPO*

Le domande seguenti riguardano il suo capo diretto e lei come suo/a collaboratore/rice.

**NB. CAPO DIRETTO = il/la suo/a coordinatore/rice infermieristico/a, non il/la dottore/ssa che coordina il reparto in cui lei lavora.**

**14. Per ciascuna domanda, indichi quanto la descrive pensando a specifiche situazioni in cui si trova a operare come collaboratore del suo capo.**

|  |  | 0  Mai | 6  Sempre |  |
| --- | --- | --- | --- | --- |
| 1 | Quando inizia un nuovo lavoro o incarico, lei ottiene subito una serie di successi che siano importanti per il suo capo? | ⓪ ① ② ③ ④ ⑤ ⑥ | |  |
| 2 | Prende l’iniziativa per trovare e portare a termine con successo incarichi che superano e vanno oltre il suo lavoro? | ⓪ ① ② ③ ④ ⑤ ⑥ | |  |
| 3 | Pensa e sostiene in maniera indipendente nuove idee che potrebbero significativamente contribuire agli obiettivi del suo capo o dell’organizzazione? | ⓪ ① ② ③ ④ ⑤ ⑥ | | |
| 4 | Cerca di risolvere i problemi complessi (tecnici o organizzativi), piuttosto che aspettare che il suo capo lo faccia per lei? | ⓪ ① ② ③ ④ ⑤ ⑥ | |  |
| 5 | Ha l’abitudine di discutere internamente l’adeguatezza delle decisioni del suo capo piuttosto che fare semplicemente quello che le viene detto? | ⓪ ① ② ③ ④ ⑤ ⑥ | |  |
| 6 | Quando il suo capo le chiede di fare qualcosa che va contro alle sue preferenze personali o professionali, dice “no” invece che “sì”? | ⓪ ① ② ③ ④ ⑤ ⑥ | |  |

|  |  | 0  Mai | 6  Sempre |
| --- | --- | --- | --- |
| 7 | Agisce in base ai suoi standard etici piuttosto che in base agli standard del suo capo o del gruppo? | ⓪ ① ② ③ ④ ⑤ ⑥ | |
| 8 | Sostiene il suo punto di vista sulle questioni importanti anche se questo potrebbe significare un conflitto con il suo gruppo o ritorsioni dal suo capo? | ⓪ ① ② ③ ④ ⑤ ⑥ | |

**Grazie molte per il tempo che ha dedicato alla compilazione del questionario!**
